# Diagnosis and evaluation of elderly-onset myasthenia gravis: A case report

**DOI:** 10.1097/MD.0000000000036989

**Published:** 2024-01-26

**Authors:** Takako Tachikawa, Shinichi Takeshima, Nobuyuki Kawate

**Affiliations:** a Department of Rehabilitation Medicine, Showa University School of Medicine, Kanagawa, Japan; b Department of Internal Medicine, Sakuragaoka Central Hospital, Kanagawa, Japan

**Keywords:** dysphagia, elderly-onset myasthenia gravis, extraocular muscle symptom, ice pack test, pyridostigmine

## Abstract

**Rationale:**

Patients with elderly-onset myasthenia gravis can have a good prognosis with appropriate diagnosis and response, although it is difficult to differentiate between exacerbations of myasthenia gravis in elderly patients and age-related changes. Therefore, it is important for physicians to understand the clinical characteristics and safe assessment methods for patients with elderly-onset myasthenia gravis.

**Patient concerns:**

An 82-year-old male diagnosed with myasthenia gravis 6 months prior had no difficulty in daily living. After falling on a golf course, he was diagnosed with a right femoral neck fracture on the 1st day and underwent right total hip replacement surgery on the 12th day, being transferred to our hospital for rehabilitation therapy on the 32nd day. However, immediately after transfer, the patient showed fatigability during training and difficulty swallowing food.

**Diagnoses:**

This case was diagnosed as an exacerbation of myasthenia gravis.

**Interventions:**

Pyridostigmine was initiated with the expectation of immediate effect on the 54th day.

**Outcomes:**

His symptoms and physical functions improved immediately, and walking distance and food intake increased. From this clinical course, it was judged that immunosuppressive therapy was indicated as a transition to generalized myasthenia gravis. For this reason, he was discharged after arranging postdischarge visits to the department of neurology, accordingly.

**Lessons:**

A better understanding of the characteristics of elderly-onset myasthenia gravis may allow for relatively safe assessment of the condition and improve its diagnosis and treatment.

## 1. Introduction

Myasthenia gravis (MG) is an autoimmune disease characterized by clinical manifestations such as fatigable weakness of ocular, bulbar, respiratory, and limb muscles, brought on by autoantibodies acting on various components of the postsynaptic membrane.^[1]^ MG patients are classified based on the type of autoantibodies detected in their serum, including antibodies against acetylcholine receptors (AChR), muscle-specific tyrosine kinases, and lipoprotein-related protein 4.^[1]^ Recently, an increasing number of elderly-onset cases (age of onset >65 or >70 years) have been reported in various countries,^[2–4]^ including Japan.^[5]^ The prognosis for patients with elderly-onset MG has been considered poor because of the numerous complications and changes in pharmacokinetics, which limit the application of aggressive treatment strategies.^[3]^ Lately, however, elderly-onset MG has been shown to be more responsive to treatment than cases with earlier onset <65 years of age^[6]^ and even low-to-moderate doses of steroids appear to be sufficiently effective.^[5]^ Thus, the pathogenesis of elderly-onset MG remains unclear. Because of it, it is necessary to diagnose and detect MG exacerbations based on the symptoms and course of the disease in elderly patients to promptly provide them with specialized medical care. About 20% of elderly patients with cerebral small vessel disease, however, have dysphagia, even though they are apparently healthy,^[7]^ and it is difficult to distinguish in some cases between this and dysphagia caused by MG. In other words, it is not easy to distinguish between an exacerbation of MG symptoms and age-related changes, which can make reaching a diagnosis difficult.

Physical and emotional burden is considered one of the exacerbating factors for MG in general,^[8]^ furthermore. The rehabilitation treatment itself may become a physical and emotional burden for the elderly patient, leading to the onset or exacerbation of latent MG in the convalescence ward, which offers intensive rehabilitation. Here we report on a case in which MG exacerbation was identified during rehabilitation treatment at the convalescence ward, after which the patient was referred to specialized care. We believe that presenting a relatively safe method of assessing MG symptoms through the characteristics of this case might help improve the diagnosis of MG in the elderly since patients with older-onset MG can have a good functional and life prognosis with appropriate diagnosis and response.

Because we considered him competent enough to make decision, we provided a sufficient explanation to the patient in question and obtained written consent from him for this report.

## 2. Case presentation

### 2.1. History of present illness

An 82-year-old male fell while moving on a golf course and was diagnosed with a right femoral neck fracture, which determined the need of a surgery. However, as he had been diagnosed with MG at 6 months ago and the risk of exacerbation from the use of anesthetics, a careful preoperative examination was conducted. On the 12th day, right total hip replacement surgery was performed under spinal anesthesia. After a prolonged period of bed rest and insufficient improvement in physical function, he was transferred to our hospital on the 32nd day for rehabilitation.

### 2.2. Past medical history and comorbidities

He had an angina and was taking low-dose aspirin after percutaneous coronary stenting. In addition, he had diabetes mellitus. He was under ongoing treatment with antidiabetic agents after temporary preoperative insulin administration.

An anterior mediastinal tumor had been detected 6 years prior. Ten months ago, he noticed right ptosis and fatigue at evening. Four months later, he was diagnosed with ocular form of MG based on high anti-AChR antibody levels (13.3 nmol/L vs normal value <0.3 nmol/L) and low-frequency stimulation of median, accessory, and facial nerves with waning on repetitive nerve stimulation test. At the time, a treatment plan including thymectomy was proposed but the patient did not agree; thus, his MG remained untreated. He was capable of independently performing activities of daily living and caring for his wife with dementia despite his condition.

### 2.3. Condition on admission

Unrestricted eye movements but no ptosis. The voice was loud and there was no salivary retention. Thoracic movements were stable and there was no fatigue. Upper extremity muscle strength was preserved while lower extremity muscle weakness was observed with a manual muscle testing of approximately 3 in the proximal muscles.

He could turn in bed and get up with light assistance, sit and stand up independently by grasping a support, and walk about 20 m with a walker under supervision with respect to the activities of daily living, at the time of admission.

### 2.4. Findings on admission

**Figure 1.**
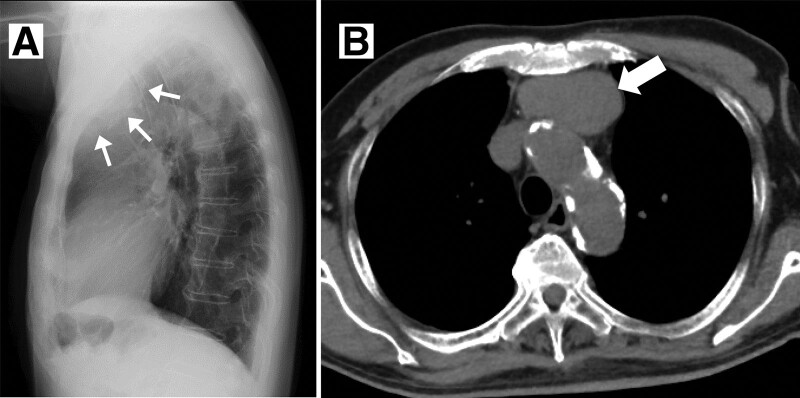
(A) Lateral view of chest X-ray. A mass lesion (arrow) from the ascending aorta to the aortic arch. (B) Computed tomography of the chest revealing a well-defined mass lesion (arrow) of 60 mm in the anterior mediastinum. The mass lesion tended to increase; thus, a noninvasive thymoma was suspected.

The white blood cell count was 6220/μL (neutrophils 62.7%, lymphocytes 28.5%, monocytes 5.8%, eosinophiles 2.4%, basophiles 0.6%), hemoglobin was 13.3 g/dL, and platelet count was 24.2 × 104/μL. In addition, the levels of various blood indicators were: total protein 7.7 g/dL, albumin 3.3 g/dL, creatinine 1.42 mg/dL, BUN 26.2 mg/dL, AST 21 U/L, ALT 6 U/L, LDH 213 U/L, ALP 182 U/L, CRP 0.34 mg/dL, blood glucose level 111 mg/dL, HbA1c 6.1%, TSH 3.92 μIU/mL (normal 0.61–4.23 μIU/mL), free T4 1.56 ng/dL (normal 0.90–1.70 ng/dL), and anti-AChR antibody 10.7 nmol/L (5th day). There were no findings other than complete right bundle branch block in the electrocardiogram. A mass extending from the ascending aorta to the arterial arch was observed in the lateral view of the chest radiography (Fig. 1A) and in chest computed tomography (1st day), a well-defined mass of about 60 mm in size was detected in the anterior mediastinum (Fig. 1B).

**Figure 2.**
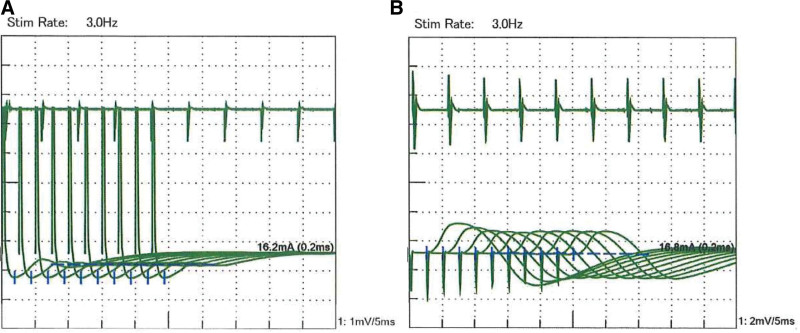
Repetitive nerve stimulation test. Decrease of 15% to 20% in M wave amplitude detected with orbicularis oculus and trapezius muscle stimulation. (A) Orbicularis oculus; (B) trapezius muscle.

Repetitive nerve stimulation test (at the time of MG diagnosis) with low-frequency stimulation at 3 Hz showed a 15% to 20% decrease in M wave amplitude of facial and accessory nerves (Fig. 2) and 10% to 40% decrease in M wave negative area of facial, accessory, and median nerves.

### 2.5. Clinical course

He was diagnosed with Myasthenia Gravis Foundation of America Severity I due to his unremarkable MG symptoms at the time of admission.

However, he fatigued easily and showed a decrease in lower limb muscle strength to an manual muscle testing of 2 during the training intervention at our hospital (Table 1). Therefore, it was necessary to adjust the walking distance while estimating fatigue from the degree of hip flexion during walking during gait training.

**Table 1:** Clinical manifestations associated with myasthenia gravis during our hospital.

Clinical manifestations during our hospitalization	
Conversation	Normal
Volume of voice	Normal
Mastication	Fatigue even with soft foods
Swallowing	Frequent coughing
Breathing	Shortness of breath during movement
Grooming	Independence
Getting up from a chair	Upper limb support required
Double vision	Without self-awareness
Ptosis	Without self-awareness
Gait	Stable
Gait distance	Fluctuates within a day

Fatigable weakness of limbs and dysphagia became to be prominent although extraocular muscle symptoms were barely noticeable.

In addition, his food intake decreased due to difficulty swallowing (Table 1) and his weight decreased. There was salivary retention and decreased sputum output, considered symptoms of worsening swallowing function; thus, a video fluoroscopic examination of swallowing was performed on the 39th day. During the 10 minutes examination, an increase in food residuals in the vallecula of epiglottis and a decrease in pharyngeal contractility were detected over time. He had complications of diabetes mellitus and ischemic heart disease, and it was difficult to explain the fluctuating easy fatiguability in a unified way, although the possibility of stroke, including cerebral small vessel disease, as a cause of dysphagia was considered.

We suspected the exacerbation of MG from the above. Therefore, 120 mg/d of pyridostigmine was started on the 54th day; its effect on easy fatigability during gait training and dysphagia resulted in decreased complaints of fatigue during the afternoon training time and increased walking distance from 30 to 60 m. The residual amount in the vallecula of epiglottis decreased and he no longer easily fatigued during the time of examination, compared to before treatment at a video fluoroscopic examination of swallowing on the 60th day. Based on these changes in clinical symptoms, pyridostigmine was considered to be effective.

We concluded accordingly that the clinical course of this case was due to an exacerbation of MG based on the transition from ocular MG to generalized MG with symptoms of bulbar palsy, which required specialized therapeutic intervention. It was best for him to be treated at hospital since the family could not provide assistance and given his low awareness of the disease and risk of developing crisis from aspiration. However, he refused to stay in the hospital for a long time and strongly desired to be discharged. Therefore, he was discharged on the 76th day after proper environment at home was set and that he would visit the department of neurology after discharge. At the time of discharge, his physical function was mostly independent in indoor activities of daily living, but he required assistance in instrumental activities of daily living, such as outdoor transportation and housework, due to easy fatigability.

It was suspected an exacerbation of MG based on medical history and symptoms not directly related to the femoral neck fracture such as easy fatigability and dysphagia in this case. Furthermore, his response to pyridostigmine led to a diagnosis, and it was able to connect him to specialized treatment before he became severe.

## 3. Discussion

### 3.1. Background and characteristics of elderly-onset MG in Japan

MG is a disease that can develop at any age, from childhood to adulthood and clinically classified into ocular MG, in which symptoms are limited to extraocular muscle symptoms, and generalized MG. Clinical characteristics of patients with elderly-onset MG include lower likelihood of complete remission to treatment^[3]^ and worse outcome in case of exacerbation, including death.^[9,10]^ In contrast, a Japanese survey on the clinical characteristics and treatment strategy of patients with elderly-onset MG showed that most patients had ocular MG, and even if they had generalized MG, treatment showed that a higher percentage achieved minimum manifestation status with prednisolone ≤5 mg/d compared to cases with onset <65 years of age.^[5]^ Accordingly, the possibility of MG should be considered in elderly patients presenting with suggestive symptoms, and good outcomes can be expected with appropriate diagnosis and treatment, even with a severe condition with crisis and dysphagia.^[6]^ However, symptoms such as easy fatigability, muscle weakness, slurred speech, and dysphagia may be diagnosed as stroke^[11]^ or aging changes in elderly patients with many comorbidities so the diagnosis of MG in elderly patients may be underdiagnosed.^[12]^

### 3.2. Clinical characteristics and evaluation of patients with elderly-onset MG

Over 50% of patients with ocular MG transition to generalized MG within the first 2 years after disease onset.^[13]^ Despite a tendency for the disease, especially in untreated cases, to shift to generalized MG within a year,^[14]^ the risk factors for this shift are still unclear. On the other hand, causes of MG exacerbations include physical and emotional stress, respiratory infections, drugs (succinylcholine, vecuronium, etc), surgery, and trauma.^[8,15,16]^

We think that this case transitioned from ocular to generalized MG within 1 year of onset because his thymoma remained undiagnosed due to refusal of thymectomy, and no therapeutic intervention for MG since diagnosis. However, we assume that the fall that triggered the fracture was not directly related to MG symptoms since there were no significant problems in his daily life and his symptoms associated with MG worsened rapidly after intensive rehabilitation began. In other words, it is possible that MG symptoms may have been exacerbated by the physical stress related to the overload of walking itself due to the muscle weakness caused by pain and postoperative disuse, as well as the expansion of training hours per day in the convalescence ward.

In the elderly, the eyelid area decreases with age due to the weakening of the small muscles around the eye and sagging skin.^[17]^ This makes it difficult to distinguish between age-related changes and ptosis. In addition, age-related changes such as macular degeneration and cataracts reduce the visual field and are less likely to be noticed as diplopia, making it easier to overlook extraocular muscle symptoms caused by MG in the elderly compared to younger patients.^[17,18]^ In this case, the previous diagnosis of MG facilitated to suspect the easy fatigability and dysphagia observed immediately after admission as symptoms of a neuromuscular junction disorder, even without identifying the extraocular muscle symptoms. In addition, the response to pyridostigmine further supported this possibility.

Based on the above, the detection of extraocular muscle symptoms is particularly important for diagnosing latent MG in the elderly. Although we did not perform them in this case, the fatigability test on sustained up-gaze and the ice pack test are relatively simple bedside assessments of extraocular muscle symptoms. The fatigability test on sustained up-gaze determines the appearance or exacerbation of obvious ptosis after the patient is allowed to gaze upward for up to 1 minute. In the ice pack test, a frozen ice pack is placed on a closed eyelid for about 2 minutes to check its effect on ptosis. The eye fissure is measured before and after the ice pack test, which is considered positive if there is improvement in ptosis of ≥2 mm. The ice pack test has also been reported to have higher sensitivity than the repetitive nerve stimulation test and equal or better specificity in diagnosing MG.^[16]^ As these tests do not require special drugs and allow for safe evaluations, they are highly valuable screening tools for elderly-onset MG.

On the other hand, the edrophonium test is a pharmacological test for MG diagnosis that can be performed at the bedside. However, it is difficult to perform in the elderly and patients with heart failure because it is associated with serious side effects such as bradycardia, loss of consciousness, and respiratory failure, in addition to gastrointestinal symptoms.^[19]^ Pyridostigmine is the most commonly used oral treatment for MG; its efficacy is supported by electrophysiology, with unlikely cholinergic side effects such as abdominal pain, diarrhea, bradycardia, and hypotension if administered at ≤300 mg/d.^[20]^ Accordingly, pyridostigmine administration is recommended instead of the edrophonium test as adjunctive diagnosis.^[3]^ As in the present case, a small dose of pyridostigmine for short-term evaluation can be administered relatively safely in the convalescent rehabilitation ward. However, pyridostigmine is prone to cholinergic side effects with age^[21]^ and long-term administration has been reported to cause degeneration of the postsynaptic fold at nerve endings in the red muscle in rats.^[22]^ Therefore, pyridostigmine should be used only at the introduction of MG treatment, and immunosuppressive therapy should be started promptly.^[3]^

The methods of evaluating patients with MG presented in this study have several limitations, including the following. First, the use of the fatigability test on sustained up-gaze or ice pack test may be limited, since 22% of MG patients admitted to the hospital do not have ptosis.^[16]^ Second, patients with muscle-specific tyrosine kinases antibody-associated MG respond poorly to pyridostigmine so they may not respond to small doses.^[20]^ Thus, these methods alone might not be sufficient to evaluate some MG patients.

## 4. Conclusions

Response to small dose of pyridostigmine and identification of extraocular muscle symptoms might allow relatively safe evaluation of latent MG in elderly patients with unexplained dysphagia or fatigability not directly related to the underlying disease.

## Acknowledgments

The authors would like to express their deep appreciation to Kayo Takeshima for her great effort in preparing this report.

## Author contributions

**Data curation:** Takako Tachikawa, Shinichi Takeshima.

**Writing – original draft:** Takako Tachikawa, Shinichi Takeshima.

**Conceptualization:** Shinichi Takeshima.

**Writing – review & editing:** Shinichi Takeshima.

**Project administration:** Nobuyuki Kawate.

**Supervision:** Nobuyuki Kawate.
